# Evaluation of Iodine Supplementation in Pregnant Women with Gestational Diabetes: IODIAB Study

**DOI:** 10.3390/healthcare10122388

**Published:** 2022-11-28

**Authors:** Dured Dardari, Francois-Xavier Laborne, Caroline Tourte, Elodie Henry, Alfred Penfornis

**Affiliations:** 1 Diabetology Department, Centre Hospitalier Sud Francilien, 91000 Corbeil-Essonnes, France; 2 Laboratoire de Biologie de l’Exercice pour la Performance et la Santé LBEPS, Université Paris Saclay, 91025 Evry, France; 3 Unité de Recherche Clinique, Centre Hospitalier Sud Francilien, 91000 Corbeil-Essonnes, France; 4 Paris-Sud Medical School, Paris-Saclay University, 91100 Corbeil-Essonnes, France

**Keywords:** pregnancy, gestational diabetes, iodine supplementation

## Abstract

Background: Iodine supplementation is indicated by the French National Authority for Health (HAS) and the World Health Organization (WHO) during pregnancy. This study investigates whether this supplementation is consistently prescribed in line with WHO recommendations in pregnant women diagnosed with gestational diabetes mellitus. Method: A total of 99 women with a diagnosis of gestational diabetes were included in the study and were all closely monitored. Results: Only 17 (17.2%) patients received the recommended iodine supplementation. The follow-up, whether conducted by a gynecologist or midwife, did not influence the prescription of iodine supplements. By contrast, 72 (72.7%) of patients received folic acid supplementation. Conclusions: The prescription of iodine supplementation for the pregnant women included in our study is insufficient. Few practitioners seem aware of the recommendations, even when the pregnancy is complicated by gestational diabetes.

## 1. Introduction

Pregnancy is characterized by elevated serum thyroxine-binding globulin concentrations, as well as increased renal blood flow and glomerular filtration, leading to increased iodide clearance from plasma, and thus loss of iodine. Increased type 3 deiodinase activity in the placenta increases the degradation of thyroxine to the inactive reverse triiodothyronine [1]. Thyroid hormones are essential for neurodevelopment both in utero and early in life [2], with severe iodine deficiency causing a range of disorders, such as hypothyroidism, goiter, and growth and neurodevelopmental impairment [3]. It is well established that correcting severe iodine deficiency leads to better clinical outcomes, including reduced rates of congenital hypothyroidism and lower infant mortality rates [4]. Recently, there have been increasing concerns that even mild-to-moderate iodine deficiency leads to adverse clinical outcomes, including potentially lower intelligence quotients (IQ) in offspring [5,6]. 

The physiological requirements for iodine are higher in pregnant women, increasing from 150 to 250 μg/day. Iodine intake is assessed by the iodine concentration in urine, and the target level of urinary iodine is between 150 and 250 μg/L for pregnant women [7]. In France, the general population is not iodine-deficient, according to the 2006–2007 National Nutrition Health Study, with median urinary iodine concentrations of 136 μg/L in adults aged 18 to 74 years [8,9]. However, according to the French studies of Raverot et al. [10], Luton et al. [11], and Hieronimus et al. [12], the median urinary iodine of pregnant women ranged from 50 to 100 μg/l, which suggests moderate iodine deficiency. To maintain normal thyroid function, a mother must increase her production of thyroxine by 40–50%, which requires an additional daily iodine intake of 150–250 μg [13,14,15]. Although the thyroid contains a reserve of iodine, this is depleted during pregnancy without supplementation. The World Health Organization (WHO), United Nations of International Children’s Emergency Fund, and International Council for Control of Iodine Deficiency Disorders recommend iodine intake of at least 250 µg/day during pregnancy. This supplementation should be facilitated by adding 150 µg iodine to all multivitamins and mineral supplements intended for pregnant women [16]. 

According to the WHO’s definition, gestational diabetes mellitus is a disorder of carbohydrate tolerance, leading to hyperglycemia of varying severity, which is diagnosed for the first time during pregnancy. Pregnant women with gestational diabetes benefit from close follow-up and monitoring.

This study aimed to explore whether iodine supplementation of 250 μg/day was prescribed to pregnant women with gestational diabetes who benefit from close monitoring following the WHO recommendations. The primary evaluation criterion was, thus, daily iodine supplementation prescribed in μg. A secondary objective was to evaluate iodine supplementation according to the prescriber: midwife, family physician, or gynecologist.

## 2. Materials and Methods

### 2.1. Study Approval

The study protocol was approved by the French National Agency for Medicines and Health Products Safety (ANSM) and by the ethics committee of Agneres University Hospital (Institutional Review Board; Agreement of Department of Health and Human Services No. RCB: 2020-A02570-39). Comprehensive information on the study was provided to each patient in a document printed for this purpose. The study was registered on ClinicalTrials.gov with the number NCT04683211.

### 2.2. Participants 

The inclusion criteria in the study were pregnant women with gestational diabetes confirmed by abnormal fasting glucose levels or high oral glucose tolerance test values, with an age ≥ 18 years, and providing informed consent. Exclusion criteria prohibited the involvement of those with existing thyroid pathology.

### 2.3. Data Collection 

Upon arrival at hospital for the management of gestational diabetes, the information note was given to patients. During the consultation, the investigator explained the study and obtained the patients’ consent to participate. The questionnaire was then given to patients. This study did not include any patient follow-up. Participation involved completing the questionnaire with the following questions: number of weeks of gestation, number of pregnancies, chronic diseases and their treatment, and known thyroid disease and its therapy. Regarding iodine supplementation, the questionnaire asked whether the patient had received a prescription for iodine supplementation since the start of pregnancy, and, if so, which supplement, along with the prescribed duration and dose. In the case of prescribed iodine supplementation, the prescriber was noted as the midwife, attending family physician, or gynecologist. The questionnaire also asked whether the patients received folic acid during this pregnancy and whether they took iodine and vitamin supplements during their previous pregnancies.

### 2.4. Statistics

For each quantitative parameter, descriptive analysis includes the mean, standard deviation, median, interquartile, minimum, and maximum. Qualitative parameters were expressed as numbers and corresponding percentages. 

We hypothesize that one in two patients received iodine supplementations of 250 μg/day. As a result, the sample size required was 97 patients.

If p is the expected percentage, its confidence interval of 95% is calculated as follows:

p ± 1.96 × √(p × (1 − p)/n).

To calculate the distance (d) between the estimate of the percentage and the bounds of its confidence interval, the following equation is calculated:

d = 1.96 × √(p × (1 − p)/n)

We also introduce this equation:

d = [1.96]^2 × p × (1 − p)/d^2 = 97 patients

The analysis was performed using R software (R version 3.5.0 (23 April 2018)© 2018, the R Foundation for Statistical Computing).

## 3. Results

The study included 99 patients. At the time of inclusion, the mean age was 31.41 [4.95] years, with a mean gestational age of 26.87 [4.39] weeks. All participants presented with gestational diabetes that was confirmed by fasting blood sugar levels of >5.06 mmol/L or by high oral glucose tolerance test values. 

Only 17 out of 99 patients (17.2%) received iodine supplementation. The mean iodine dose prescribed for this group was 200 μg/day. By contrast, 72 (72.7%) of women received folic acid supplementation at a dose of 0.4 mg/day. Overall, 16 patients (16.2%) were prescribed both iodine and folic acid. Of the 99 patients in the study, only 4 (4%) declared that they received iodine supplementation during their previous pregnancies. 

Overall, 71 (71.7%) women had been monitored by a gynecologist since the start of pregnancy, with only 13 receiving iodine supplementation (18.3%). Furthermore, 50 women (50.5%) were monitored by both a midwife and a gynecologist, with only 8 (16%) receiving iodine supplementation at the time of their inclusion in the study. Finally, 17 (17.2%) women were monitored by their family physician and a gynecologist, with 7 patients (41.1%) receiving iodine supplementation. Consequently, the group of patients monitored by their family physician from the start of their pregnancy until inclusion received the most iodine supplementation. The results of the IODIAB study are summarized in Table 1.

Only 13 patients (20.6%) of patients who reported being monitored by a hospital health practitioner received iodine supplementation.

## 4. Discussion

Iodine supplementation during pregnancy is recommended for all women by the WHO. The main objective of the IODIAB study was to assess whether the recommended prescription of this supplement is followed by the midwives, family physicians, and gynecologists who monitor pregnant women diagnosed with gestational diabetes. Our findings showed that only 17.2% of women received iodine supplementation, far below the recommended levels.

A systematic review of trials conducted over the past 30 years [17] indicated that iodine supplementation during pregnancy can improve the iodine status of pregnant women and their neonates. According to this recent meta-analysis [17], the impact of iodine supplementation on maternal and neonatal thyroid parameters is reduced if the administration of iodine is delayed. Given the increasing evidence demonstrating the importance of preconception supplementation with iodine, our study, despite being non-interventional, highlights the extent to which the health practitioners who monitor women with gestational diabetes do not follow the international and national recommendations regarding iodine supplementation. As the use of iodine supplementation can increase the presence of gestational diabetes [18], the 99 women in the study managed their gestational diabetes in the same hospital but with different practitioners. When the prescription of iodine supplementation is carried out, it seems to be in line with the recommendations. Other trials have shown that the dose received by pregnant women is well above the indicated dose [19].

## 5. Conclusions

Our study sheds light on the low numbers of health practitioners who prescribe iodine supplementation to pregnant women with gestational diabetes. These findings are in sharp contrast to folic acid supplementation, which was taken by 72 (72.7%) women included in our sample.

## Figures and Tables

**Table 1:** Patient characteristics and iodine supplementation.

Variable-Mean [Standard Deviation]/N (%)	Overall Population-N=99	Iodine Supplementation		*p*-Value
		NO-N=82	YESi-N=17	
Age	31.41 [4.95]	31.51 [5.28]	30.94 [2.95]	0.54
Gravitity				0.59
1	31 (31.3)	23 (28)	8 (47.1)	
2	34 (34.3)	28 (34.1)	6 (35.3)	
3	18 (18.2)	15 (18.3)	3 (17.6)	
4	11 (11.1)	11 (13.4)	0 (0)	
5	1 (1)	1 (1.2)	0 (0)	
7	3 (3)	3 (3.7)	0 (0)	
8	1 (1)	1 (1.2)	0 (0)	
mean [standard deviation].	2.33 [1.44]	2.46 [1.51]	1.71 [0.77]	0.42
Pregnancy term	26.87 [4.39]	26.71 [4.57]	27.65 [3.35]	0.16
**Follow-up (raw variables)**				
hospital	63 (63.6)	50 (61)	13 (76.5)	0.28
gynecologist	71 (71.7)	58 (70.7)	13 (76.5)	0.77
general practitioner	17 (17.2)	10 (12.2)	7 (41.2)	**<0.01**
midwife	50 (50.5)	42 (51.2)	8 (47.1)	0.8
Iodine dose (micrograms/day)	200 [0]	--	200 [0]	--
Iodine supplementation in prior pregnancies	4 (4)	4 (4.9)	0 (0)	--
Folic acid supplementation	72 (72.7)	56 (68.3)	16 (94.1)	**0.04**
Folic acid dosage (mg/day)	0.4 [0]	0.4 [0]	0.4 [0]	1
Dual supplementation	16 (16.2)	0 (0)	16 (94.1)	**<0.0001**
